# Exploring the synthesis of mouse cursor tracking and drift diffusion modeling in a perceptual decision-making task

**DOI:** 10.3758/s13428-025-02805-0

**Published:** 2025-09-24

**Authors:** Oliver Grenke, Stefan Scherbaum, Martin Schoemann

**Affiliations:** Department of Psychology, Dresden University of Technology, Zellescher Weg 17, 01069 Dresden, Germany

**Keywords:** Movement tracking, Process tracing, Drift diffusion model, Decision-making, Perception, Cognitive mapping

## Abstract

Process tracing and process modeling are the two primary behavioral approaches for uncovering human decision-making processes. However, both approaches face significant limitations: process tracing offers a large and oftentimes confusing number of measures, while process modeling relies on a minimal number of comparable trials for reliable model fitting. In our study, we explore how we can combine mouse cursor tracking and the drift diffusion model (DDM) in order to both reduce the number of cursor measures and circumvent the minimal trial amount requirements of DDM fitting. One hundred three participants completed 90 trials in a random dot kinematogram (RDK). A total of 18 cursor measures were taken from the mouse cursor tracking literature and used to predict drift rate, threshold separation, and non-decision time of the DDM via partial least squares regression. Four cursor measures contributed significantly to the prediction of the DDM parameters. When reducing the available trials, these cursor measures, in combination with response time and accuracy, performed better and remained more stable in the prediction of DDM parameters than model fitting. Our results lower the barrier for applying mouse cursor tracking for novice researchers by highlighting important cursor measures and their mapping to psychological constructs of decision-making, while also offering an approach for behavioral scientists to investigate DDM components in experimental setups with a restricted number of trials.

## Introduction

In the behavioral study of human decision-making processes, two approaches are commonly employed: first, measuring choice frequencies as well as response times (RTs) and analyzing the resulting distributions applying a process model, often a diffusion model (Ratcliff, 1978); second, measuring data from the decision process itself (i.e., process tracing), such as, via mouse cursor or eye tracking (Schulte-Mecklenbeck et al., 2017).

Process models are fitted to choice and RT distributions and yield central parameters offering intuitive, psychological interpretations. The drift diffusion model (DDM), for instance, yields as its core parameters the *drift rate*, *threshold separation*, *non-decision time*, and *starting bias,* which intuitively map on distinct cognitive components of the decision-making process in two-alternative forced-choice tasks. Evidence is accumulated toward one of two response thresholds, with or without a preexisting bias in favor of one response (starting bias). The distance in the evidence space between the thresholds is described by the threshold separation. Noise is added to the evidence accumulation (drift rate), which allows the model to account for a variety in choices and RTs. If the evidence accumulation surpasses either threshold, the respective response is selected and the RT is computed by adding the accumulation time to the stimulus processing and motor execution times, which together form the non-decision time (for a review, see Ratcliff & Smith, 2004). Due to the model’s dependency on fitting distributions, a minimum number of trials per experimental condition and per subject is required to reliably estimate its parameters (Voss et al., 2013). The minimal required number of trials, however, depends on fitting algorithms and other boundary conditions (Lerche et al., 2017). For example, for the fast-dm-30 algorithm (Voss & Voss, 2007, 2008) using the maximum likelihood estimation, it is recommended to use more than 40 trials (Voss et al., 2013) and fast-dm-30 does not allow fitting with fewer than ten trials. This requirement limits the application of process models to empirical scenarios that offer a high enough number of trials across all conditions.

In contrast, process tracing offers a more direct access to the distinct cognitive components of the decision-making process in every single trial in an experiment. Mouse cursor tracking lends itself very well to the study of the decision-making processes in two-alternative forced-choice tasks (for reviews, see: Freeman, 2018; Schoemann et al., 2021; Stillman et al., 2018). It yields time-continuous measures (e.g., time-continuous multiple regression; Scherbaum & Dshemuchadse, 2020) or summary measures (e.g., area under the curve; Spivey et al., 2005) which, as the name implies, summarize different aspects of the cursor trajectory (from here on referred to as *cursor measures*). Typical cursor measures encompass the deviation from an optimal trajectory, complexity in movement, as well as temporal aspects of the cursor movement (Wirth et al., 2020; Wulff et al., in press). Given the relative youth of the field, the number of cursor measures is still growing, resulting in a widespread diversity of measures for a number of components of the decision-making process. Such a range of different measures is susceptible to two issues: First, it leaves open questions of convergent and divergent validity of the different measures and the associated components of the decision-making process, which is evident in a significant correlational overlap within and between categories of measures (Wulff et al., in press); second, it may pose an intimidating hurdle for scientists aiming to adopt mouse cursor tracking but lacking the overview to select appropriate measures for their decision-making component of interest.

In this article, we combine process modeling and process tracing in order to pursue two aims: First, to functionally categorize and thereby reduce the number of cursor measures by mapping cursor measures to parameters of the DDM. Second, to reduce the number of trials necessary to extract components of the decision-making process by using the categorized cursor measures from these trials instead of fitting a DDM. To this end, we conducted an exploratory, cursor-tracking study employing a simple two-alternative forced-choice decision-making task. The implemented task aimed at creating a close connection between empirical and modeled behavior as captured by the DDM (for more details, see sections “Apparatus, stimuli, and procedure” and “Limitations and future research”) and hence enable the matching of model parameters and cognitive components. We investigated the following exploratory research questions:

- **RQ1:** Can cursor measures be mapped onto parameters of the DDM in order to provide a correspondence between each cursor measure and distinct cognitive components of the decision-making process?
- **RQ2:** To what extent can the number of mapped cursor measures be reduced to a small core set of measures in order to lower the hurdles for the application of mouse cursor tracking?
- **RQ3:** To what extent can we reduce the number of trials on which the cursor measures are based and can this reduction undercut the number of trials needed to fit the DDM?

## Methods

### Participants

We recruited 103 participants (63 female, median age = 24 years, range = 18–73 years). All participants had normal or corrected-to-normal vision and gave separate informed consent.

Two tasks were completed, from which only one will be analyzed in this study due to its limited scope. The task order was balanced across participants. The experiment required approximately 15 min and was appended to the data collection of three different, unrelated studies. The sample size was thus restricted by the time and monetary resources of the other studies. Participants were recruited through an online recruiting system for psychological studies at Technische Universität Dresden and through direct contact with psychology faculty members between March and October 2023. Overall study time was kept below 90 min. Payment of participants in cash, vouchers, or class credits was dependent on the studies the tasks were appended to and ranged from 15 to 20 EUR.

### Apparatus, stimuli, and procedure

The experiment was run in MATLAB 2018b on a Windows 10 personal computer using a 23.8-inch Fujitsu Display B24-8 TE Pro (1920 × 1080, 60 Hz, positioned approximately 60 cm from participants), a Logitech G413 keyboard, and a high-precision computer mouse (Logitech G203 Prodigy Gaming Mouse) and Psychophysics Toolbox 3.0.15. Cursor trajectories were sampled with a frequency of approximately 107 Hz from the presentation of the stimulus material until a response had been carried out. The mouse cursor speed was set to 4/11 of the maximum operation system’s setting while the proprietary Logitech Software LCore was set to 800 dpi. This resulted in a cursor/mouse-movement ratio of around 4.26. Non-linear acceleration (“Enhance pointer precision” option in Windows) was disabled.

**Fig. 1:**
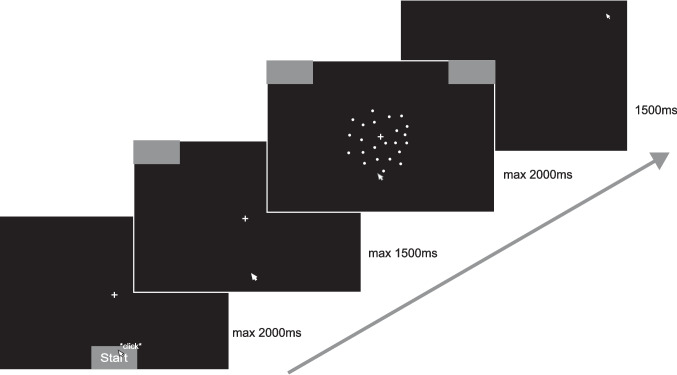
Task setup. *Note.* Phase order from left to right: start phase, pre-stimulus phase, stimulus phase, inter-trial phase. Phase duration is printed to the right of the phase screen. Participants had to click with the mouse cursor into the start box at the bottom of the screen. After clicking, response boxes appeared in the upper corners of the screen, and participants had to initiate cursor movement upwards in order to start the trial. After reaching a movement threshold, the stimulus material appeared, and participants had to move the mouse cursor to the left or the right response box according to the RDK movement or arrow direction. After responding, an inter-trial screen was presented

The study consisted of a single coherence task in which participants were asked to decide on each trial in which directions (left or right) a random-dot kinematogram (RDK) was moving (Fig. 1). The RDK is well established in the context of sequential sampling models. This can be traced back to evidence in single-cell recordings in which the firing patterns in macaque monkeys while solving an RDK task resembled an accumulation-to-threshold process (Roitman & Shadlen, 2002). Since then, the RDK has been implemented in studies of human decision-making in combination with the DDM (Ratcliff & McKoon, 2008). The RDK consisted of 100 white dots against a black background with a diameter of 6.5 px. The dots were presented in a round aperture with a diameter of 13° visual angle (approx. 500 px), centered on the screen. In each frame of the RDK, 6% of the dots were randomly selected as target dots moving coherently in the direction to be identified^1^; the remaining non-target dots moved in random directions (Brownian Motion algorithm; Pilly & Seitz, 2009). The spatial displacement for every dot for each apparent movement was 1 px. The displacement rate was 30 Hz, which results in a speed of 30 px/s. Due to the display in use this equals 0.79°/s (30 px). The movement of the RDK was linked to the movement of the cursor, so that the apparent RDK movement would stop if participants stopped moving the cursor. This served to encourage continuous computer mouse movements (see Appendix A  Table A1 for the specifics of this coupling).

Each RDK trial was split into four parts (Fig. 1): start phase, pre-stimulus phase, stimulus phase, and inter-trial phase. Participants were required to start a trial within 5 s, complete movement initiation in the pre-stimulus phase within 1.5 s, and terminate the stimulus phase within 2 s. The inter-trial phase lasted 1.5 s. If any time limit was reached, the trial was aborted. Feedback in the inter-trial phase informed the participants that they had reached the time limit, and, unbeknownst to them, the trial was restarted. During the start phase, participants were required to click on the start button (white "Start" in Times New Roman in 24 pt; 140 × 60 px) situated at the center bottom of the screen. In start, pre-stimulus, and stimulus phase, a white fixation cross was depicted in the center of the screen. In the pre-stimulus phase, two grey response boxes (224 × 100 px) appeared in the upper left and right corners. In order to trigger the stimulus onset, participants had to initiate cursor movement by moving the mouse upwards (i.e., dynamic starting procedure; Scherbaum & Kieslich, 2018). The onset distance was jittered across trials, randomly sampled from a uniform distribution between 53 and 152 px to minimize stimulus onset expectations. In the stimulus phase, the RDK was presented and participants moved the cursor in either one of the response boxes to indicate their response (i.e., hover response procedure; Schoemann et al., 2019). RTs were measured as the duration of the stimulus phase. Finally, in the inter-trial phase, participants moved the cursor back to the position of the start box and received feedback of time limit breaches if appropriate.

Participants completed 90 experimental trials. Before processing these trials, participants completed an automated step-by-step tutorial as well as 20 practice trials, half of which were with and without time limits, respectively. In addition to time limit feedback, participants received response feedback (correct/incorrect – Times New Roman in 24 pt). The feedback for correct practice trials was depicted in green, and all other feedback in yellow. At the end of the tutorial, participants were reminded by the experimenter to move the mouse without stopping and to be as accurate and fast as possible. Across all trials, we balanced and randomized the movement direction of the target dots between left and right.

### Data preprocessing and statistical analyses

Analyses were run in MATLAB 2020a (preprocessing, DDM fitting, fit assessment, & cursor measure extraction) and with R version 4.3.1 (R Core Team, 2023) in RStudio (Posit team, 2023; fit assessment, correlational reduction, PLS regression, resampling procedure). In R, we mainly used *{mvtnorm}* (*v1.2.2*; Genz & Bretz, 2009), {*plsVarSel}* (*v0.9.10*; Mehmood et al., 2012), and *{pls}* (*v2.8.2*; Liland et al., 2023) for the statistical modeling; the correlation matrix was computed with *{hmisc}* (*v5.1.1*;Harrel Jr, 2023); visualization was done with *{corrplot}* (*v0.92*; Wei & Simko, 2021) and {*ggplot2}* (*v3.5.1*; Wickham, 2016).

### DDM fitting

In order to enhance the fit of the DDM to the RT distributions of correct and erroneous trials, we excluded outliers. For early outliers, we discarded the earliest 3.5% of trials per participant to avoid biased fit. For the late outlier, we removed all trials with an RT > 2 standard deviations (per participant). This procedure resulted in an overall removal of 691 (7.45%) of all trials, which left 8579 trials for all subsequent analyses.

We used the fast-dm-30 (Voss & Voss, 2007, 2008) algorithm to compute a parameter set per participant. The parameter sets consist of three out of the four basic DDM parameters: *drift rate*, *threshold separation*, and *non-decision time*. The starting bias was set to a neutral position, as the movement direction of the target dots was balanced and randomized across trials.

In order to assess our empirical fits, we simulated choices and RTs based on 1000 synthetic parameter sets (Voss et al., 2015). These parameter sets were drawn from a three-dimensional normal distribution on the basis of the covariance matrix of the fitted, empirical model parameters. As a next step, each synthetic data set was fitted via fast-dm-30 with identical settings to the empirical fitting procedure. Crucially, the synthetic RT distributions of correct and erroneous choices were limited in the same way as our empirical data: We selected only RTs below 2 s and drew 90 data points at random. The distributions of the synthetic fits were used to assess the fit quality of the empirical fits: Empirical fits below the 5% quantile of the synthetic fit distributions are considered bad fits (Voss et al., 2015). In our case, no empirical parameter set was detected as having a bad fit.

### Trajectory preprocessing and cursor measures

We only used the raw cursor trajectories of the stimulus phase. All trajectories were centered to the same starting position on the *x*-axis, and trajectories leading to the right were mirrored to the left. We then extracted all cursor measures (Table 1) and computed the mean of the cursor measures per participant to achieve comparability with the DDM parameters.

**Table 1:** Extracted cursor measures

Cursor measures name	Short description	Source
Acceleration changes (accChanges)	Number of times the mouse cursor acceleration changes signs	Cranford & Moss, 2018
Average deviation (AD)	Average deviation from an optimal trajectory	Wulff et al. in press
Area under the curve (AUC)	Area between optimal and actual trajectory	Wirth et al. 2020
Curvature	Length of actual trajectory divided by length of optimal trajectory	Wirth et al. 2020
Maximum absolute deviation (MAD)	Maximum distance from the actual trajectory to the optimal trajectory	Wirth et al. 2020
Maximum absolute deviation above the optimal trajectory (MADabove)	Same as MAD except only distances towards the alternative response option are considered	Wulff et al. in press
Maximum acceleration (maxAcc)	Maximum acceleration	Wulff et al. in press
Maximum velocity (maxVel)	Maximum velocity	Wulff et al. in press
Mean velocity (meanVel)	Mean velocity	-
Minimum velocity (minVel)	Minimum velocity	-
Minimum X (minX)	Minimal distance to alternative response in *x*-coordinates	Wirth et al. 2020
Motor pauses	Time in seconds in which the mouse cursor was not moved	Wulff et al. in press
Motion time	Time in seconds in which the mouse cursor was moved	Wulff et al. in press
Reversals	Number of times the cursor traversed the midline of the screen	Wulff et al. in press
Sample entropy for *x*-coordinates (sampleEnX)	Complexity measure for *x*-coordinates of cursor trajectory	Wirth et al. 2020, Hehman et al., 2015
Time to peak acceleration (timeToPeakAcc)	Time in seconds until the maximum acceleration in trajectory is reached	Wirth et al. 2020
Time to peak velocity (timeToPeakVel)	Time in seconds until the maximum velocity in trajectory is reached	Wirth et al. 2020
xFlips	Number of times the movement direction in x-coordinates changes	Wirth et al. 2020

*Note.* Overview of extracted cursor measures, with a short description and source. Measures without source are basic measures that were added by the authors; for all cursor measures from the literature that were not implemented, see Table 3

## Results

### Descriptive results

The mean accuracy of participants for the RDK task was about 78% with a range of 49–95%. Over all participants, the mean RT was 1.27 s, ranging from 0.23 to 2 s. Hence, the results suggest a sufficient variety in inter-individual performance and response behavior for subsequent analyses.

In the following, we report how we prepared the functional association between cursor measures and DDM parameters by correlational reduction of cursor measures, the analyses of the functional association via partial least square regression (PLSR), and finally the reduction of the number of trials on which the association resides.

### Correlational reduction of cursor measures

**Fig. 2:**
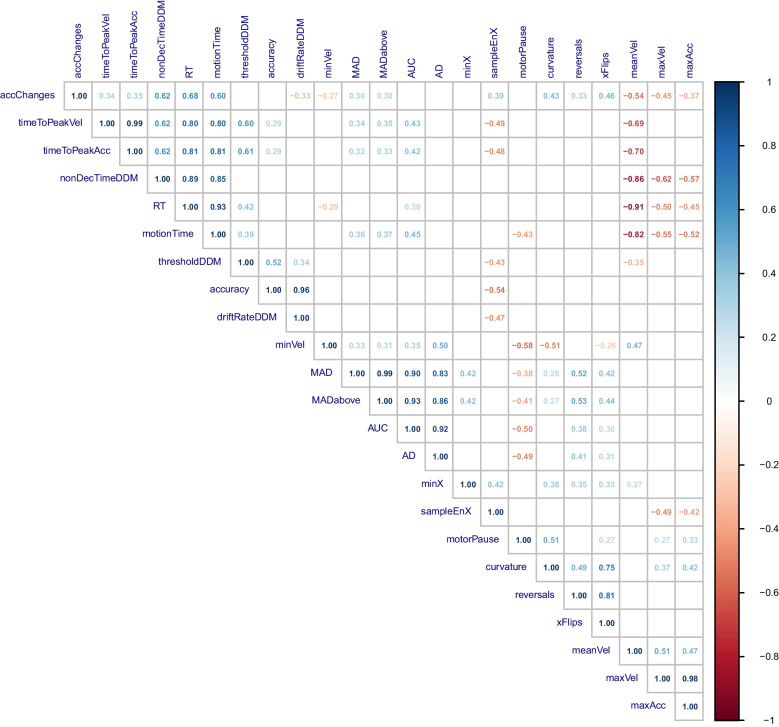
Correlation matrix of cursor measures, DDM parameters, RT, and accuracy. *Note.* Correlation matrix of cursor measures, DDM parameters, as well as RT and accuracy, rounded to the second decimal. Only significant correlations (p <.05) are depicted, and correlation strength is color-coded from 1 (blue) to – 1 (red). The variables have been clustered using hierarchical clustering

To prepare the analyses of functional association between cursor measures and DDM parameters, we investigated the correlational structure of cursor measures, DDM parameters, and classical behavioral measures (i.e., RT and accuracy). Figure 2 shows the matrix of significant (*p* <.05) correlations of these variables. We identified several clusters with highly correlated cursor measures. In order to reduce redundancies (RQ2) prior to PLSR analyses, we decided to condense these clusters to those measures that are calculated most efficiently. Thus, we excluded measures based on very high correlation (|*r*| ≥ 0.9) and retained the measure that requires fewer steps to be computed (Table 2). RT and accuracy were excluded from the analyses concerning RQ1 and RQ2 (see partial least-squares regression, PLSR) since they constitute the basis of the DDM fit.

**Table 2:** Discarded cursor measures

Cursor measure name	Very high correlation with
timeToPeakAcc	timeToPeakVel
meanVel	RT
motionTime	RT
AD	AUC
AUC	MADabove
MADabove	MAD
maxAcc	maxVel

*Note.* Excluded cursor measures due to very high correlations (|*r*| >=.9). The left column shows cursor measure name and the right column lists the remaining cursor measures

### Partial least squares regression (PLSR)

In order to establish the functional association between the remaining cursor measures and DDM parameters (RQ1) and further reduce the amount of cursor measures (RQ2), we employed partial least squares regression (for a review, see Abdi, 2010). The PLSR model is a statistical model with which predictors are clustered into independent components. In contrast to a principal component analysis, these components maximize the explained covariance of predictors and criterion variable rather than the variance of the predictors. The PLSR components (or latent variables) are then used in a linear regression. The PLSR enables researchers to estimate the predictive value of each individual predictor for the criterion variable in the presence of multicollinearity (Abdi, 2010).

To address RQ1, we implemented a separate initial PLSR model predicting each of the three DDM parameters based on the remaining cursor measures. To address RQ2, we added two further reduced model specifications to each of the initial PLSR models. All reported model performances (explained variance, *R*^2^) are results of leave-one-out cross-validation (LOO-CV).

**Fig. 3:**
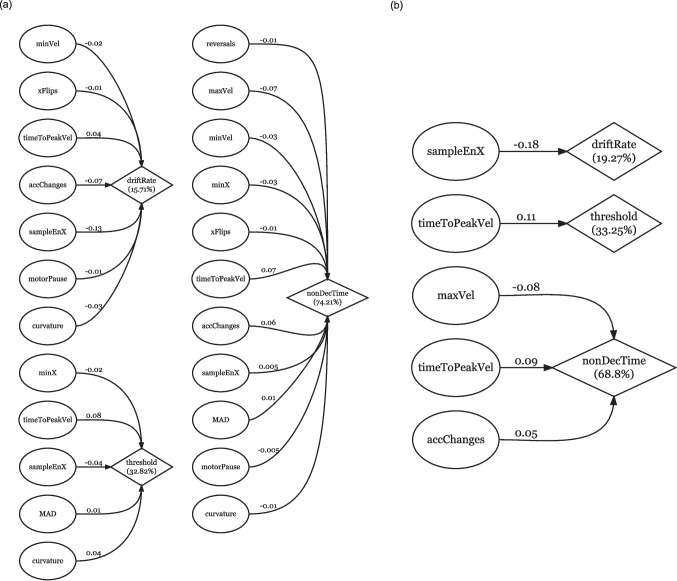
Reduced and minimum PLSR models: Explained variance and regression coefficients. *Note.* Criterion variables with cross-validated *R*^2^ (diamonds), predictor variables (circles) with PLSR coefficients for (**a**) reduced PLSR models and (**b**) minimum PLSR models. Predictors (circles) included in (**a**) displayed matching signs between PLSR and correlation coefficients. Predictor variables in (**b**) contributed most to the explained variance as determined by VIP and further reduction on the basis of only minimal loss in *R*^2^ and model fit

For the initial models, we followed a stepwise procedure and discarded those predictors for which regression and correlation coefficients did not match in sign, since such predictors are not interpretable in a psychologically relevant way (for a short discussion of multicollinearity, see Daoud, 2017). If multiple measures did not match in sign, we discarded the measure correlating the lowest with the criterion variable, reran the PLSR, and iterated until no mismatches could be detected and a consistent initial model was identified (Fig. 3a). These initial models give us a first insight into whether cursor measures can be mapped to DDM parameters. Crucially for RQ1, cursor measures were able to explain part of the variance for each examined DDM parameter: *drift rate* (15.71%), *threshold separation* (32.82%), and *non-decision time* (74.21%). In order to reduce the initial models in accordance with RQ2, we selected predictors on the basis of their explicatory contribution to the respective model. We first computed the variable importance in projection (VIP; Chong & Jun, 2005; Mehmood et al., 2012) then discarded all predictors with a VIP value lower than 0.8, and finally iteratively discarded further predictors with VIP values lower than 0.8 as long as the model fit (*root mean square error of prediction* – RMSEP - of the LOO-CV) did not decrease (Andersen & Bro, 2010). These *VIP-reduced PLSR models* (Appendix Fig. A1) were further reduced by probing which predictors could be discarded with minimal loss in explained variance and model fit. This last step only resulted in a reduction for the drift rate model (Δ*R*^2^ = −0.65%, ΔRMSEP = +0.001) and the threshold separation model (Δ*R*^2^ = −0.87%; ΔRMSEP = +0.001)^2^. With the minimal PLSR models, we arrived at four cursor measures, that offer the best explanatory power for our fitted DDM parameters (Fig. 3b). Drift rate is best explained (19.27%) by sampleEnX. Threshold separation is best explained (33.25%) by timeToPeakVel*.* Non-decision time is also best explained (68.8%) by timeToPeakVel, as well as maxVel and accChanges.

### Trial reduction resampling

**Fig. 4:**
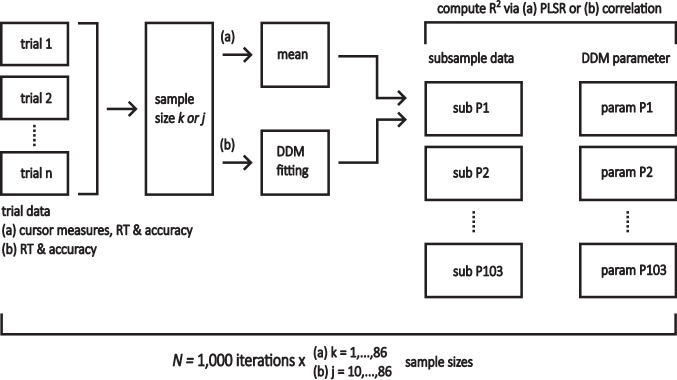
Pipeline of the resampling procedure*. Note.* Resampling procedure for (**a**) PLSR models and (**b**) subset DDM parameters. The resampling followed a bootstrapping procedure with 1000 iterations per sample size. Sample size was varied from (**a**) 1 or (**b**) 10 to the maximum trial size in all participants after outlier correction. For each resulting *R*^2^ distribution, the mean and 95% confidence intervals were computed

In order to reduce the number of trials on which the cursor measures are based (RQ3), we implemented a resampling procedure. Here, we iteratively computed the *R*^2^ of PLSR models for each DDM parameter, stepwise decreasing the number of trials from which the predictors were extracted and averaged (Fig. 4, pipeline a). In order to gain insight into the incremental value of using cursor measures as predictors, we included additional models that consisted of RT and accuracy (RT/AC) only, or RT, accuracy, and the respective cursor measures of the minimal PLSR models (hybrid). Each PLSR run was repeated 1000 times per trial sample size. The resampling followed a bootstrapping procedure in the sense that cursor measures were drawn with replacement for each repeated PLSR run; DDM parameter, however, remained fixed. In order to contrast and to compare the thus produced R^2^ distributions, we additionally fitted DDM models to accuracy and RT distributions sampled with the same approach described above and correlated the resulting DDM parameter with their respective counterpart of the complete data set, on the basis of which we computed the respective *R*^2^ values (Fig. 4, pipeline b).

**Fig. 5:**
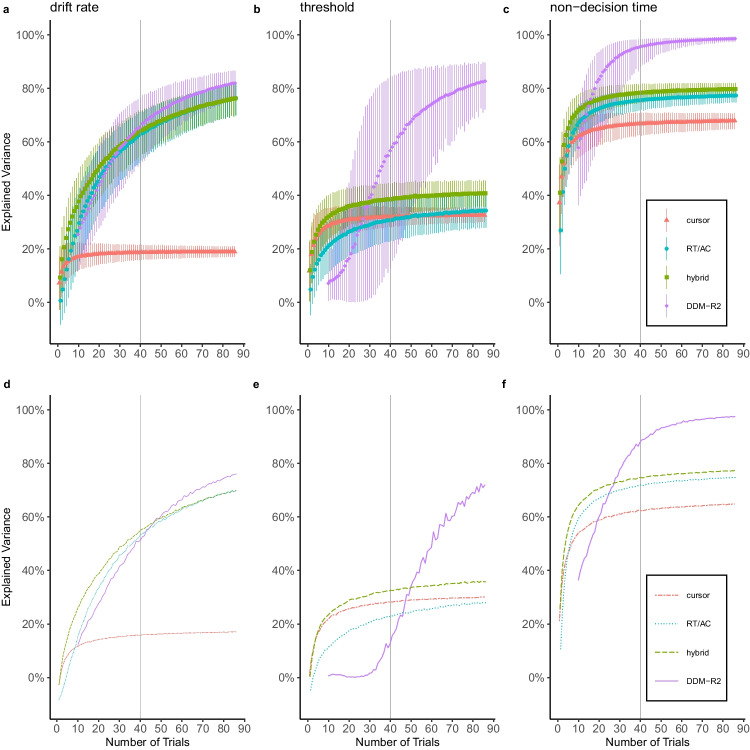
Results of the resampling procedure. *Note.* Resulting cross-validated *R*^2^ for (**a, d**) drift rate, (**b, e**) threshold separation, and (**c, f**) non-decision time; (**a–c**): Mean and 95% confidence interval of the cross-validated *R*^2^ for each resampled model; cursor measures of the minimal PLSR models (triangle), RT and accuracy (dot), cursor measures, RT, and accuracy (square) and *R*^2^ of parameter self-correlation (diamond). (**d–f**): lower boundary of the 95% confidence interval for each resampled model. The vertical line depicts the recommended minimum amount of 40 trials for using fast-dm-30 (Voss et al., 2013)

This second resampling gave us an estimation of the stability for each DDM parameter when fitting is based on a diminishing number of trials and offered us a basis on which we assess the stability of PLSR model estimates. Both resampling procedures differed in the number of sample sizes due to the limited minimum sample size of 10 trials allowed for fitting the DDM by fast-dm-30. The results are shown in Fig. 5. Crucial for RQ3, PLSR models retained explanatory power well below the 40-trial mark; especially for threshold separation and non-decision time, the hybrid models showed a greater stability in comparison to the DDM self-correlation. For the drift rate, hybrid PLSR models performed marginally better than the self-correlation. Non-decision time models retained explanatory power down to a single trial.

### Trial order effect

**Fig. 7:**
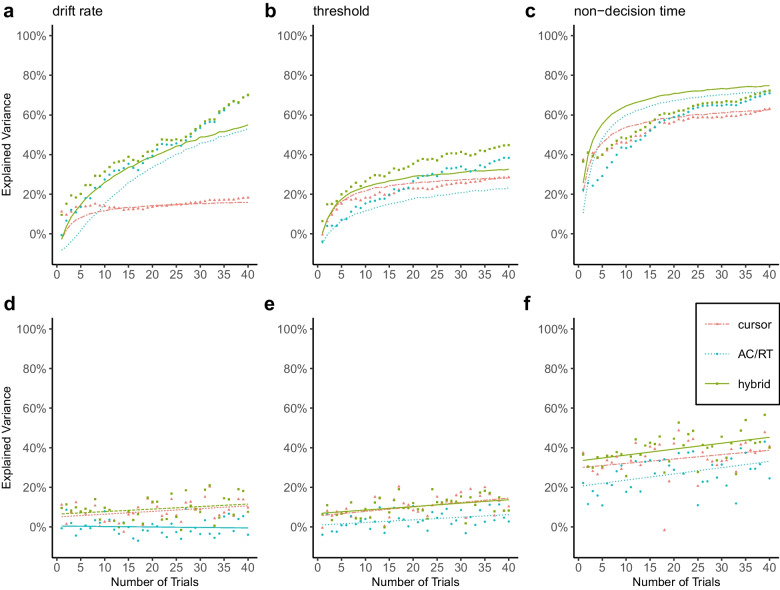
Accumulated and single-trial PLSR models. *Note*. Analyses of trial order effects of the PLSR models for (**a, d**) drift rate, (**b, e**) threshold separation, and (**c, f**) non-decision time. The maximum number of trials is limited to 40, as this constitutes the minimum recommended trial amount for the used DDM. (**a–c**) Comparison between accumulated *R*^2^ (shapes) and lower boundary of the resampled PLSR models’ 95% confidence intervals (lines). (**d–f**): scatter plots with linear regression of single-trial correlations. The number of trials is not identical with trial number due to outlier exclusion

To investigate how much insight the cursor measures from a reduced number of trials can provide, we performed control analyses for trial order effects. If the participant’s behavior, and thus the cursor measures, only stabilizes after executing a certain number of trials within the main experiment, reducing trials below this number would not be warranted even if we find stable results for a lower number of trials later in the experiment. For this reason, we additionally computed the same PLSR models (cursor, RT/AC, hybrid) while retaining the trial order: First, we computed the PLSR models based on the accumulation of trials (first trial, first and second trial, etc.; Fig. 7a–c). Second, we computed the PLSR models per single trial for the first until the last available trial (Appendix Fig. A2d–f). The accumulated *R*^2^ of the non-decision time models fall mostly below the 95% confidence interval of the resampled mean. However, no PLSR model showed major trial order effects by losing all explanatory power for fewer trials.

## Discussion

In this exploratory study, we combined two approaches to measure decision-making processes, the drift diffusion model (DDM) as a process model and mouse cursor tracking as a process tracing method. We investigated the functional association between cursor measures and DDM parameters and their implications based on three research questions (RQ1, RQ2, and RQ3). Overall, we found that changes in cursor measures capture changes in all DDM parameters, and that the number of cursor measures can be reduced to four distinct measures. Furthermore, we found that the number of trials on which the cursor measures are based can be significantly reduced, thereby undercutting the number of trials needed to fit the DDM, especially for threshold separation and non-decision time.

In the following, we first focus on our exploratory research questions, address limitations and future research directions, before we draw a final conclusion.

*Can cursor measures be mapped onto parameters of the DDM in order to provide a correspondence between each cursor measure and distinct cognitive components of the decision-making process? (RQ1)*

Yes, cursor measures can be mapped onto each examined DDM parameter. We found that 15.71% of the variance in the drift rate, 32.82% of the variance in the threshold separation, and 74.21% of the variance in the non-decision time can be explained with the maximal amount of predictors in interpretable PLSR models. There is, however, still a large overlap in the contribution of cursor measures across DDM parameters, rendering a clear correspondence to distinct cognitive components of the decision-making process difficult. Hence, a better functional understanding of the cursor measures is provided by answering RQ2.

*To what extent can the number of mapped cursor measures be reduced to a small core set of measures in order to lower the hurdles for the application of mouse cursor tracking? (RQ2)*

From the 18 included measures, there are four measures that contribute a major part to the explained variance in our analysis. For drift rate, sampleEnX contributed most explanatory power (19.27%). For threshold separation, timeToPeakVel contributed most explanatory power (32.82%). For non-decision time, three measures contributed most explanatory power, namely maxVel, timeToPeakVel, and accChanges (68.8%). With this reduction, we determined which cursor measures can be mapped onto which distinct cognitive components of the decision-making process (RQ1). However, we could not establish an exclusive one-to-one mapping between cursor measures and components, because timeToPeakVel maps onto both threshold separation and non-decision time.

The mapping of sampleEnX to the drift rate follows the existing literature. SampleEnX, as a sophisticated measure of the spatial complexity of the trajectory’s movement on the *x*-axis, has been attributed to competition of response alternatives (Dale et al., 2007; Hehman et al., 2015). A higher competition between response alternatives should hence be associated with a slower evidence accumulation, which can be seen in our data in the correlation of sampleEnX and drift rate (*r* = –.47). Therefore, a highly complex *x*-trajectory can be compared to a very noisy evidence accumulation. It should be noted, however, that the explained variance for the drift rate was at a low level. This is astonishing given the presence of well-established cursor measures meant to capture differences in performance, such as AUC and MAD. An obvious problem is the lack of correlation with drift rate, which can be attributed to a missing association between cursor measures and accuracy, as accuracy and drift rate correlated very highly. As we extracted and used cursor measures both from correct and incorrect trials, we departed from previous approaches of excluding erroneous trials (Spivey et al., 2005). In order to verify our approach, we computed a correlation matrix with correct trials only (Appendix Fig. A3): The correlational landscape did not change meaningfully, especially not with respect to drift rate. Another reason for the overall poor explanatory power for the drift rate may be a difference in task design: Curvature measures such as MAD or AUC have often been employed in within-subject designs (e.g., Koop & Johnson, 2013; Spivey et al., 2005), whereas our study focuses solely on between-subject differences. We will address this issue in future research.

The mapping of timeToPeakVel to threshold separation seems surprising, considering the literature. To date, timeToPeakVel has mainly been analyzed in studies examining movement control, planning, and adjustments in target-directed movement (e.g., Jacko et al., 2000; Stewart et al., 2013). Hence, it is typically conceptualized as a descriptive measure of continuous action control, which appears to be unrelated to the psychological interpretation of the threshold separation as the required amount of evidence to make a decision (Ratcliff & McKoon, 2008). However, the relation becomes more plausible if we take into consideration that the threshold separation is sensitive to experimental manipulations of the speed–accuracy trade-off (Ratcliff & McKoon, 2008). Speed–accuracy trade-offs in movements do not only influence the overall speed – rather they also shift peak velocities (Plamondon & Alimi, 1997). Considering the process of responding with a computer mouse in a decision-making task, such shifts in the peak velocity seem reasonable: After finalizing the decision-making process, participants aim to log-in their response as quickly as possible, which in turn means that peak velocity is reached shortly after. If the threshold separation is low, participants require less evidence and finalize their decision quickly, and hence reach peak velocity early. In contrast, if the threshold separation is high, participants require more evidence and finalize their decision slowly, and hence reach peak velocity late. This interpretation is substantiated by a positive correlation between timeToPeakVel and threshold separation (*r* =.60).

The mapping of maxVel, and timeToPeakVel to non-decision time is in line with the existing literature: Both measures are used to describe continuous action control in target-directed movement. When participants reach their final decision in a decision-making process captured by cursor movements, the remaining trajectory can be conceptualized as the response execution aspect of the non-decision time (Ratcliff & Smith, 2004) which may be associated with the time point of maxVel. If maxVel is high, this corresponds to a lower non-decision time, as the response is executed quickly; this is the case in our data (*r* = –.64). Following this logic, it appears contradicting, that a short timeToPeakVel is not associated with a longer but rather a shorter non-decision time (*r* =.62) as one would expect a longer response execution period following an earlier decision. However, in our data, shorter timeToPeakVel is associated with higher mean velocities (*r* = –.70), which explains the flip in the former association as participants had an overall reduced response time when timeToPeakVel was short (*r* =.80). The mapping of accChanges onto non-decision time comes as a surprise: AccChanges has been used to characterize the temporal complexity of cursor trajectories in the context of linguistic processing (Dale & Duran, 2011). This conceptualization places accChanges closer to components of decision-making, such as drift rate. In our data, however, its unique contribution to the drift rate was only marginally compared to sampleEnX, which is why we discarded it for the minimum PLSR models. Rather than being an indication for higher evidence accumulation, fewer accChanges can be interpreted as a more efficient movement of the computer mouse overall, which leads to lower non-decision times (*r* =.62).

*To what extent can we reduce the number of trials on which the cursor measures are based, and can this reduction undercut the amount of trials needed to fit the DDM? (RQ3)*

The cursor measures of the minimal PLSR models demonstrated a surprisingly high level of stability in their mapping onto components of the decision-making process for trial numbers lower than the minimal trial requirement for fitting DDM parameters. Hybrid PLSR models performed well for low numbers of trials and remained predictive even below the 10-trial threshold, which constitutes the technical minimum of the fast-dm-30 fitting algorithm. PLSR models based only on cursor measures performed worse throughout the study. This is not surprising as RT and accuracy are the basis of the DDM fitting and are therefore highly associated with its parameters. However, the cursor measures show increased model performance, especially for low trial amounts, demonstrating the incremental value of cursor measures. This highlights the potential of implementing process tracing methods in empirical scenarios where trial numbers are limited to those below the minimum trial requirement for fitting DDM parameters, although trial order effects should be considered.

## Limitations and future research

Here we want to address four limitations of our study.

First, even though our results encourage further research into the use cases for mouse cursor measures, this is a first exploratory study. However, we took care that all PLSR models were cross-validated with a leave-one-out procedure. We can therefore be optimistic that the performance of the models is not overestimated. Nevertheless, a confirmatory study will be necessary to fully substantiate the here-defined models and results.

Second, presumably the most striking result, concerns the rapid loss in stability of the self-correlation of threshold separation in the simulation, even before the 40-trial mark. At first glance, this might be a problem of the model fit and the data did not lend itself to being fit with the drift diffusion model. This is, however, unlikely, as we tested the quality of our empirical model fit with an elaborate procedure by which we created a synthetic set of DDM fits (Voss et al., 2015). None of our empirical model fits fell below the 5% threshold of synthetic fits (see Data preprocessing and statistical analyses), suggesting that the DDM describes our current data well (Voss et al., 2015). Another reason for this early instability might be a stark sensitivity of the DDM fitting procedure on the different subsamples. The maximum likelihood estimation, which is implemented as an optimization criterion for fitting small samples, is known to be greatly influenced by even single early outliers (Voss et al., 2013). A similar effect than an early outlier might have taken place when drawing the subsamples and hence different distributions in RT, which in our case almost exclusively influenced the threshold separation parameter. It is important to point out that the cursor PLSR model with timeToPeakVel, as well as its hybrid counterpart, remained more stable throughout the resampling space – making this measure a prime candidate for researchers interested in low sample size threshold separation estimation.

Third, the complexity of the study setup, that is task (single-stage perceptual decision) as well as model variation of the DDM (three basic parameters) was kept as simple as possible. Varying the task difficulty within participants might shed additional light onto cursor measures more suitable to predict the drift rate, as most cursor measures are deployed in studies using within-subject designs. Future research will demonstrate how different and more complex tasks affect the reliability of cursor measures across contexts, further deepening our understanding of which additional psychological constructs cursor measures can be mapped to. In this sense, the presented study demonstrates a framework that allows for both the simplification of mouse cursor tracking and the mapping of hand trajectories onto psychological constructs.

Fourth, we base our current work on the assumption that the DDM parameters do represent the cognitive components relevant to the decision-making behavior. This assumption is in turn based on the empirical association between the implemented task and both sequential sampling as well as the DDM itself (see sections “Introduction” and “Apparatus, stimuli, and procedure”). However, the assumption is a simplification: Not all relevant aspects of the decision-making process may be best characterized by a sequential accumulation-to-threshold process and added non-decision time. This simplification (and therefore distortion) of empirical phenomena is a general feature of models, cognitive or statistical, which should encourage viewing models as helpful tools in a wider scientific process (or to use a well-known aphorism generally attributed to George E.P. Box: “All models are wrong, but some are useful.”). This is why combining cursor measures with other modeling approaches is a promising future avenue for further understanding not only the potential and limits of mouse cursor tracking in decision-making but also the processes of decision-making in general.

## Conclusion

In this study, we combined the drift diffusion model (DDM) and mouse cursor tracking in order to pursue two aims: First, to functionally categorize and thereby reduce the number of cursor measures by mapping cursor measures to parameters of the DDM. Second, to reduce the number of trials necessary to extract components of the decision-making process by using the categorized cursor tracking measures from these trials instead of fitting a DDM. We showed that the number of cursor measures can be reduced significantly and can be mapped to components of the decision-making process, therefore lowering the implementation barrier for researchers new to mouse cursor tracking, even though an overall one-to-one mapping of cursor measures and cognitive components was not achieved. Furthermore, we showed that cursor measures combined with response time and accuracy may offer an overall better prediction and stability for a low number of trials compared to DDM fitting. The presented approach to cursor tracking opens up a new way to use and implement selected cursor measures. The detail in which cursor tracking allows analyses promises to alleviate central problems in task design for behavioral psychologists. Thus, the synthesis of mouse cursor tracking and the framework of the DDM offers a simple and parsimonious avenue for behavioral researchers interested in decision-making processes.

## Figures and Tables

**Fig. 6:**
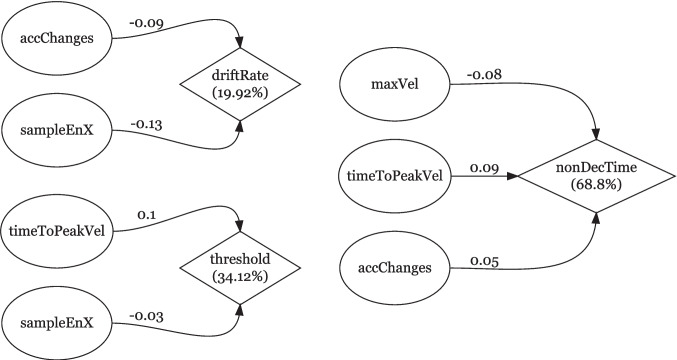
VIP reduced PLSR models: Explained variance and regression coefficients. *Note.* Criterion variables with explained variance (diamonds), predictor variables (circles) with PLSR coefficients for the VIP-reduced PLSR model. Predictors from the reduced PLSR model (Fig. 3a) were discarded on the bases of (i) a VIP value < 0.8 and (ii) further measure-by-measure reduced by discarding predictor below VIP < 0.8 if model fit did not diminish with the exclusion. In case of multiple VIP values < 0.8, the predictor with the lowest value was discarded

**Fig. 8:**
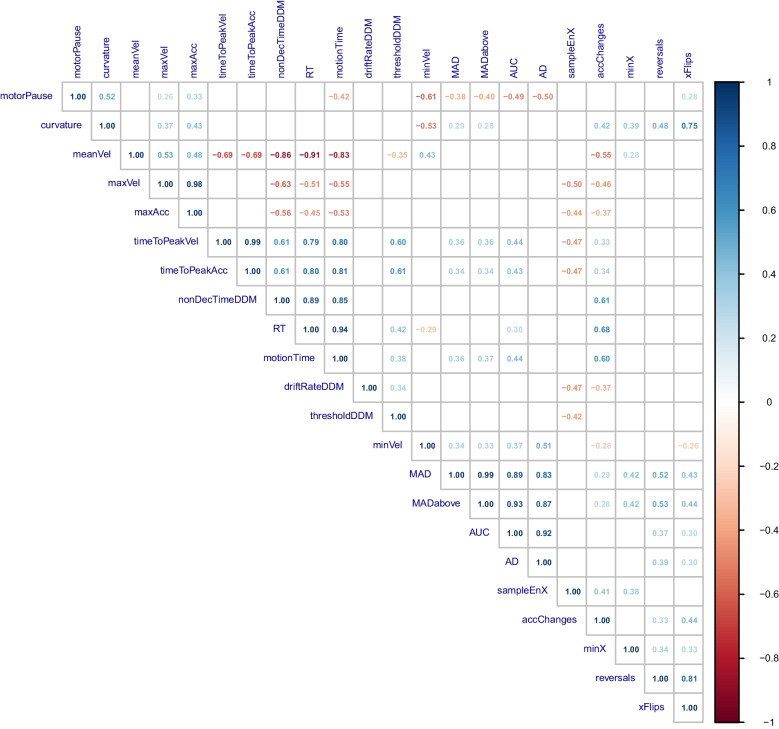
Correlation matrix of cursor measures, RT, and accuracy of correct trials only. *Note.* Correlation matrix of correct trials only for cursor measures, DDM parameters, as well as RT, rounded to the second decimal. Only significant correlations (*p* <.05) are depicted. Color scale on the right-hand side for color coding of correlation strength from 1 (blue) to – 1 (red). The variables have been clustered using hierarchical clustering

## Data Availability

All data, experimental code, and materials are available under https://osf.io/2vcdu/.

## Appendix A

**Table 3:** Not implemented cursor measures

Cursor measure name	Short description	Source	Reason for exclusion
Click time	Time interval between entering the target area and clicking	Wirth et al. 2020	No click for response
Final distance to target	Distance from click position in response box to the center of the response box	Wirth et al. 2020	No click for response
Idle time	Sum of initiation time & motor pause	Wulff et al. in press	Stimulus onset after movement initiation.
Initiation time	Time interval between the click/touch of the starting area and leaving the starting area.	Wirth et al. 2020/Wulff et al. in press	Stimulus onset after movement initiation.
Movement time	Time in seconds in which mouse cursor was moved.	Wirth et al. 2020	Identical to motion time (Wulff et al. in press)
Starting angle	Angle of the movement when leaving the starting area relative to the vertical midline.	Wirth et al. 2020	Stimulus onset after movement initiation, no dedicated starting area

*Note:* Mouse cursor measures not implemented in current study, with a short description, source, and reason for exclusion

### Coupling of mouse cursor & RDK

As the aim of sampling the mouse cursor trajectory is to identify the decision process during the trial, a continuous movement during the stimulus phase is needed to capture said process in the hand movement. We therefore encouraged participants during the display of the random dot kinematogram (RDK) to keep moving the computer mouse by coupling the RDK and mouse cursor movement. Only if certain conditions were met did the RDK’s motion continue. If the cursor was situated below the response boxes, RDK motion only occurred if participants moved the cursor upwards (i.e., in minus *y*-direction). If the cursor was situated between the response boxes, dot motion occurred only if the cursor was moved in *x*-direction, additionally depending on which half of the screen the cursor was situated: If the mouse cursor was on the left half of the screen, only movement to the left animated the RDK. Accordingly, if situated on the right half of the screen, the cursor had to be moved to the right side to view RDK motion. These specific “between box” conditions were implemented to discourage participants from alternating cursor movement between the response boxes in order to observe the RDK motion without having to commit to an answer. This kind of motion does not lend itself to the measures extraction implemented in this study.

## Footnotes

1 The coherence level of 6% was determined in a pilot study to result in an average accuracy of 80% and hence to yield potential for inter-individual variance.

2 A further reduction of the non-decision time model would have resulted in Δ*R*^2^ = – 4.64% and ΔRMSEP = + 0.007

## Code availability

All analysis code is available under https://osf.io/2vcdu/.
